# Dysautonomia in Parkinson’s Disease: Impact of Glucocerebrosidase Gene Mutations on Cardiovascular Autonomic Control

**DOI:** 10.3389/fnins.2022.842498

**Published:** 2022-03-15

**Authors:** Angelica Carandina, Giulia Lazzeri, Gabriel Dias Rodrigues, Giulia Franco, Edoardo Monfrini, Federica Arienti, Emanuele Frattini, Ilaria Trezzi, Pedro Paulo da Silva Soares, Chiara Bellocchi, Ludovico Furlan, Nicola Montano, Alessio Di Fonzo, Eleonora Tobaldini

**Affiliations:** ^1^Department of Internal Medicine, Fondazione IRCCS Ca’ Granda, Ospedale Maggiore Policlinico, Milan, Italy; ^2^Neurology Unit, Fondazione IRCCS Ca’ Granda, Ospedale Maggiore Policlinico, Milan, Italy; ^3^Centro Dino Ferrari, Neuroscience Section, Department of Pathophysiology and Transplantation, University of Milan, Milan, Italy; ^4^Department of Clinical Sciences and Community Health, University of Milan, Milan, Italy; ^5^Laboratory of Experimental and Applied Exercise Physiology, Department of Physiology and Pharmacology, Fluminense Federal University, Niterói, Brazil

**Keywords:** Parkinson’s Disease, glucocerebrosidase gene mutations, cardiovascular autonomic control, dysautonomia, heart rate variability (HRV)

## Abstract

Evidence from clinical practice suggests that PD patients with the Glucocerebrosidase gene mutations (GBA-PD) are characterized by more severe dysautonomic symptoms than patients with idiopathic PD (iPD). Therefore, an accurate assessment of cardiovascular autonomic control (CAC) is necessary to clarify the role of GBA mutations in the pathophysiology of PD. We evaluated the CAC at rest and during orthostatic challenge of 15 iPD, 15 GBA-PD and 15 healthy controls (CTR). ECG and respiration were recorded in supine position and during active standing. The analysis of Heart Rate Variability (HRV) was performed on ECG recordings using two different approaches, linear spectral analysis and non-linear symbolic analysis. GBA-PD patients presented more frequently an akinetic-rigid phenotype and cognitive dysfunction than iPD patients. Both iPD and GBA-PD group were characterized by a lower spectral HRV than CTR group. At rest, the GBA-PD group was characterized by a lower parasympathetic modulation and a shift of the sympathovagal balance toward a sympathetic predominance compared to the CTR group. Moreover, the GBA-PD patients presented a lower HR increment and a lower or absent reduction of the vagal modulation in response to the active standing than iPD patients. Lastly, the cardiovascular autonomic dysfunction in PD patients was associated with longer disease duration, and with the occurrence of REM sleep behavior disorder and constipation. Our findings suggest a more severe impairment of the CAC in PD patients with GBA mutations. These results and further studies on the role of GBA mutations could allow a stratification based on cardiovascular risk in PD patients and the implementation of specific prevention programs.

## Introduction

Parkinson’s Disease (PD) is the second most frequent neurodegenerative disorder after Alzheimer’s Disease; it affects an estimated 1.2 million people in Europe and the number is expected to double by 2030 as a result of the population ageing (Dorsey and Bloem, 2018). In the last thirty years, there have been great advancements in the understanding of the genetic background of PD. Approximately 10–15% of PD cases are associated with pathogenetic variants of the β-glucocerebrosidase gene (GBA), conferring a cumulative risk of developing PD of 5% at age 60, rising to 15–30% at age 80 (Avenali et al., 2020). It has been suggested that both a chronic loss of the lysosomal enzyme β-glucocerebrosidase (GCase) activity as well as a possible toxic gain-of-function of the mutated protein result in lysosomal dysfunction and endoplasmic reticulum stress, which could contribute to disease pathogenesis (O’Regan et al., 2017). The presence of GBA gene mutations has been associated with worse motor outcomes, with more rapid motor decline (Maple-Grødem et al., 2021), and more frequent cognitive impairment (Zhang et al., 2018). Moreover, evidence from clinical practice suggests that PD patients carrying mutations of the GBA gene (GBA-PD) may present more frequently dysautonomic manifestations and REM sleep behavior disorder (RBD) than patients with idiopathic Parkinson’s Disease (iPD; Brockmann et al., 2011; Gan-Or et al., 2016; Petrucci et al., 2020).

A very useful non-invasive and sensitive indicator of autonomic alterations is the analysis of heart rate variability (HRV), which evaluates the oscillatory components embedded into heart period and blood pressure time series. HRV represents a measure of the global capacity of the autonomic nervous system (ANS) to respond adaptively to both exogenous and endogenous stimuli. Therefore, a lower HRV is associated with autonomic impairment (Kleiger et al., 2005). To date, through the HRV analysis, alterations of the ANS and in particular of the cardiovascular autonomic control (CAC) have already been investigated in iPD patients during wake and sleep, and in other genetic forms of PD, such as cases associated with LRRK2 mutations (Kallio et al., 2000; Visanji et al., 2017; Heimrich et al., 2021). However, to the best of our knowledge, there are no studies assessing the CAC in GBA-PD patients. Moreover, since cardiovascular dysautonomia occurs before the onset of motor symptoms [8], an accurate assessment of CAC is necessary to clarify the pathophysiologic role of GBA mutations in α-synucleinopathies.

## Materials and Methods

### Study’s Design and Population

For the present case-control study, we enrolled 15 healthy controls (CTR), 15 iPD and 15 GBA-PD patients from the Neurology Unit of Fondazione IRCCS Ca’ Granda, Ospedale Maggiore Policlinico (Milan, Italy). All PD patients fulfilled the 2015 the Movement Disorder Society (MDS) Clinical Diagnostic Criteria for Parkinson’s disease (Postuma et al., 2015). The presence of GBA mutations was investigated using Next Generation Sequencing (NGS) panel. While iPD patients were negative for pathogenic variants in all genes, GBA-PD patients carried a single heterozygous GBA variant and no other mutation in the remaining analyzed PD genes. The absence of stable sinus rhythm on ECG, history of coronary artery disease, the presence of neuro- (e.g., deep brain stimulators) or cardiac stimulators, onco-hematological conditions, ongoing acute clinical conditions, pregnancy, and consent refusal were considered exclusion criteria for this study. The enrollment and recording period elapsed between April 2020 and June 2021. Every patient underwent a single assessment and each experimental session consisted of a 20-min ECG and respiratory recording, and a neurologic clinical evaluation. For CTR subjects, ECG and respiratory activity were recorded according to the same protocol. All the experimental sessions took place between 8 a.m. and 12 noon. The protocol was approved by the local Ethics Committee (Comitato Etico Milano Area 2, approval document number: 257_2020) and it was developed in accordance with the Declaration of Helsinki. All the subjects signed informed written consent prior to study participation.

### Clinical Evaluation

All patients underwent a complete neurologic evaluation, performed by a Neurologist trained in Movement Disorders. They were staged with Hohen and Yahr (H&Y) scale (Goetz et al., 2004). Patients were divided into two major subgroups, depending on the clinical motor phenotype: tremor-dominant and akinetic-rigid. To do so, the motor symptom at onset as well as the scores on UPDRS scale, part III, were evaluated, in particular for instance those regarding rest and postural tremor versus those assessing rigidity, finger and toe tapping, leg agility and global bradykinesia. For each enrolled patient, the following information were collected: age, gender, disease duration, presence of non-motor symptoms (depression, RBD, orthostatic hypotension, supine hypertension, constipation, urinary disturbs) and cognitive involvement, presence of motor fluctuations and/or dyskinesia, use of L-Dopa, L-Dopa daily dose, use of dopamine agonists, L-Dopa equivalent daily dosage (LEDD) calculated according to Tomlinson et al. (2010), presence of cardiovascular comorbidities.

### Physiological Recordings and Cardiovascular Autonomic Control Assessment

Cardiovascular recordings were performed with spontaneous breathing in the supine position for 10 min and during active standing for 10 min. All participants were asked to avoid consuming food or caffeine in the 2 h before the recording session and carrying out physical exercise the day before. ECG (lead II) and respiration through a thoracic piezoelectric belt were recorded with a sampling frequency of 1000 Hz, using an *ad hoc* telemetric system device (LAB3, Marazza Spa, Monza, ITA). All measurements were performed in a quiet and temperature-controlled room (between 22 and 24°C), and all patients had a normal body temperature during the recordings (between 35,5 and 36,5°C).

For the analysis of HRV, two segments of 250 ± 50 beats without artifacts were selected from each ECG stationary sequence, one at rest and one in the orthostatic position. Two different approaches, linear spectral analysis and non-linear symbolic analysis, were applied through a specific software (Heart Scope II, AMPS, ITA). The autoregressive model was performed to identify the spectral power in the low-frequency band (LF, bounded between 0.04 and 0.15 Hz), index influenced by baroceptive activity, sympathetic and parasympathetic modulation, and in the high-frequency band (HF, bounded between 0.15 and 0.40 Hz) synchronous with respiration, index influenced mostly by parasympathetic modulation. The LF and HF components were expressed in absolute values (ms^2^) and normalized units (LFnu and HFnu) to represent the relative amount of each component compared to the total power of the HRV spectrum. The algorithm also calculates the LF/HF ratio, which, if evaluated together with the other spectral indices, can provide indications on the relationship between the two autonomic branches (Montano et al., 2009; Billman, 2013; Heathers, 2014).

Non-linear inter-beat dynamics were evaluated on the same segments by symbolic analysis. The R-R time series was converted into a sequence of symbols that was divided into 3-beat patterns. Patterns were classified into four families: (a) 0V, patterns with no variation, all three symbols are equal (e.g., 4-4-4); (b) 1V, patterns with 1 variation, 2 consecutive symbols are equal forming a 2-beat plateau, while the remaining one is different (e.g., 2-2-5); (c) 2LV, patterns with 2 like variations, all symbols are different from the previous one and they are in ascending or descending order (e.g., 1-3-4); (d) 2UV, patterns with 2 unlike variations, all symbols are different from the previous one but not in a consequent order (e.g., 2-5-1). The percentage of the patterns 0V is a marker of cardiac sympathetic modulation and 2UV or 2LV are markers of cardiac vagal modulation (Porta et al., 2007). With respect to spectral analysis, this approach was found suitable to assess non-reciprocal changes in sympathetic and parasympathetic modulation on heart period time series both in physiological and pathological conditions, especially those characterized by low global variability (Barbic et al., 2019; Zamunér et al., 2019). Furthermore, as it is focused on short patterns in the RR interval series, this type of analysis has been proposed to be more accurate for the study of short non-linear HRV instabilities.

To evaluate the dynamic response of CAC to active standing challenge, the relative percentage increase {ΔORT% = [(HRV in orthostatic position – HRV in supine position)/HRV in supine position] *100} was calculated for each HRV index.

### Statistical Analysis

Data were analyzed using SPSS Statistics 27 (IBM, Armonk, New York, United States). The Shapiro-Wilk test was used to evaluate the normal distribution of the data. For descriptive analysis, results were expressed as absolute frequency, relative frequency, means and standard error. For the comparisons of HRV indices between GBA-PD, iPD and CTR groups at rest, one-way ANOVA for independent measures and Tukey *post hoc* test was used. The Kruskal-Wallis and the Dunn’s *post hoc* tests were applied when the normality assumption was not respected. The ANCOVA analysis, correcting for age, was used to evaluate the differences between groups in dynamic response to standing position. Partial correlation analysis, correcting for age and presence of GBA mutations, was used to estimate the relationship between HRV indices and clinical data in all the enrolled PD patients. *P* < 0.05 was considered statistically significant.

## Results

### Demographic and Clinical Characteristics of the Study Population

**FIGURE 1:**
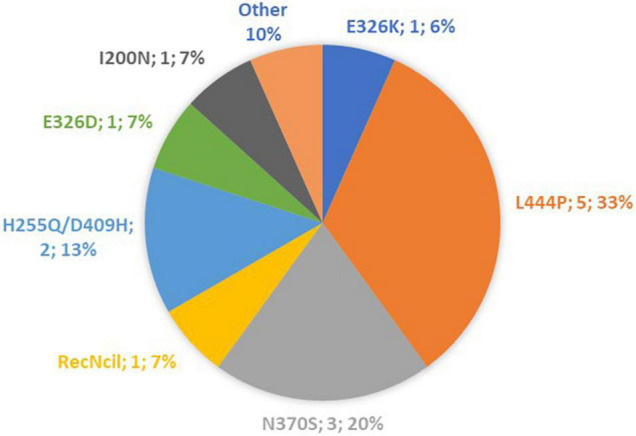
Distribution of GBA mutations in our cohort.

A total of 15 iPD patients, 15 GBA-PD patients and 15 CTR subjects were included in the present study. The distribution of GBA mutations characterizing our patient cohort is shown in Figure 1. The three groups were homogeneous for age and sex distribution (CTR group: 61.5 ± 1.6 y; 53% females. iPD 62.2 ± 2.1 y; 33% females. GBA-PD 61.1 ± 2.3 y; 40% females). The comparison of clinical features between iPD group and GBA-PD group is shown in Table 1. GBA-PD and iPD patients had comparable disease duration and H&Y score. The akinetic-rigid phenotype, evaluated by means the UPDRS part III as described above, was significantly more frequent in the GBA-PD group. With regard to motor symptoms, the iPD group and the GBA-PD group showed a comparable prevalence of postural instability, dyskinesia and fluctuations. The mean total LEDD was similar in the two groups. No significant differences emerged between the GBA and iPD groups for rivastigmine, clozapine and other anxiolytic drug assumption in terms of absolute frequencies. Since several studies highlighted an inhibitory effect of clozapine on parasympathetic cardiovascular modulation, statistical analyzes were performed to evaluate the possible difference in cardiac autonomic control in patients receiving clozapine. No significant differences were detected in HRV parameters between patients in treatment with clozapine and non-clozapine patients. No significant difference in the frequency of non-motor symptoms (NMS) was revealed. The GBA-PD group presented a higher prevalence of cognitive disfunction than the iPD group.

**TABLE 1:** Demographic and clinical features of iPD and GBA-PD patients.

	iPD (*N* = 15)	GBA-PD (*N* = 15)	*P*
			
CHARACTERISTICS	Mean ± Standard Error, n° (%)	Mean ± Standard Error, n° (%)	
Age	62.2 ± 2.1	61.1 ± 2.3	0.725
Gender (females)	5 (33%)	6 (40%)	0.705
Disease duration (years)	4.40 ± 0.74	4.67 ± 1.04	0.382
Age at onset	58.1 ± 1.9	56.6 ± 2.3	0.610
H&Y	1.67 ± 0.14	1.80 ± 0.18	0.376
UPDRS III	13.7 ± 1.2	14.6 ± 1.9	0.250
Postural instability	3 (20%)	6 (40%)	0.232
**Phenotype**			0.001*
Akinetic-rigid	3 (20%)	12 (80%)	
Tremor dominant	12 (80%)	3 (20%)	
**NMS**			
Cognitive disfunction	1 (7%)	6 (40%)	0.031*
Depression	8 (53%)	5 (33%)	0.269
Hallucinations	1 (7%)	2 (13%)	0.543
RBD	7 (47%)	11 (73%)	0.136
Symptomatic OH	3 (20%)	6 (40%)	0.231
SH	6 (40%)	3 (20%)	0.208
Constipation	8 (53%)	11 (73%)	0.256
Urinary disturbs	5 (33%)	7 (47%)	0.456
**Therapy**			
Total LEDD	557 ± 137	458 ± 92	0.467
Levodopa therapy	12 (80%)	9 (60%)	0.232
Dopamine agonist therapy	10 (67%)	10 (67%)	1.000
Clozapine	4 (27%)	8 (53%)	0.264
Rivastigmine	0 (0%)	2 (13%)	0.483
Anxiolytic drugs	2 (13%)	0 (0%)	0.483
**Complications of levodopa therapy**			
Dyskinesias	4 (27%)	3 (20%)	0.666
Motor fluctuations	3 (20%)	4 (27%)	0.666

*iPD, idiopathic Parkinson’s Disease patients; GBA-PD, Parkinson’s Disease patients with GBA mutation; H&Y, Hohen and Yahr scale; UPDRS III, Unified Parkinson’s Disease Rating Scale part III; NMS, non-motor symptoms; RBD, REM sleep behavior disorder; OH, orthostatic hypotension; SH, supine hypertension; LEDD, levodopa equivalent daily dose.*

*Significant p-values < 0.05.

*P values in bold are the significant results.*

### Cardiovascular Autonomic Control at Rest

**FIGURE 2:**
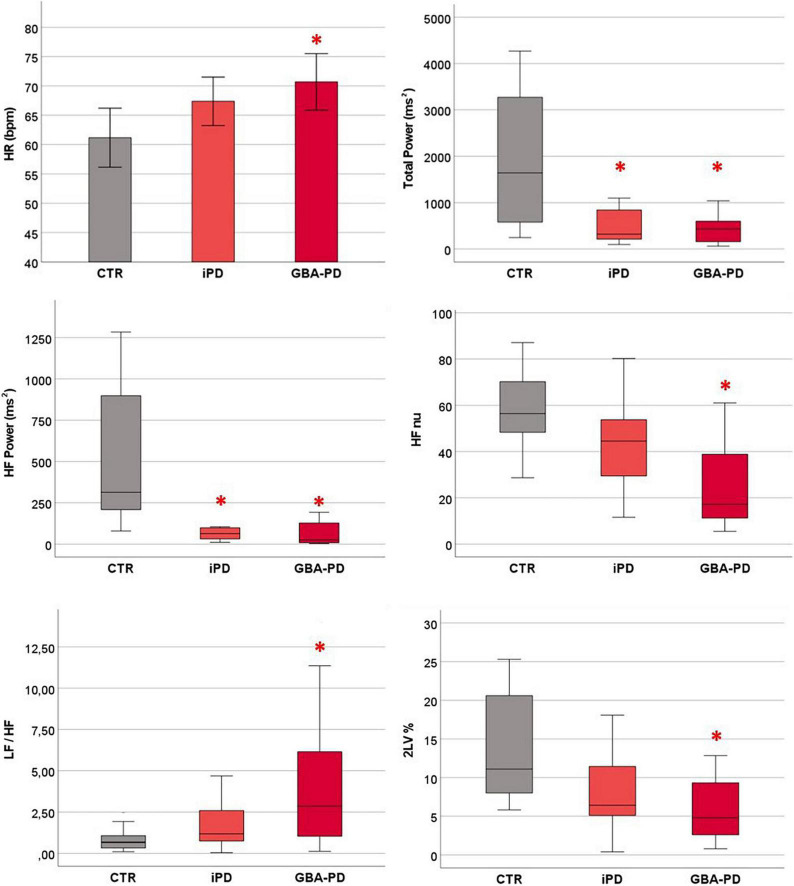
Differences between groups in cardiovascular autonomic control at rest. HR values are presented as mean ± standard error. HRV parameters are presented as median and interquartile range. CTR, control group; iPD, idiopathic Parkinson’s Disease patients; GBA-PD, Parkinson’s Disease patients with GBA mutation; HR, heart rate; HF, high frequency; nu, normalized unity; LF, low frequency power; 2LV%, patterns with 2 like variations. *Significant *p*-values < 0.05, comparison versus controls.

At rest, GBA-PD patients showed significantly higher heart rate (HR) values than CTR group (*p* = 0.02). With regard to HRV indices, the spectral analysis highlighted a lower Total Power (*p* = 0.002) in iPD and GBA-PD patients and in particular lower absolute power of the HF band (*p* < 0.001) compared to the CTR group. Moreover, parasympathetic HRV indices calculated with both the spectral (HFnu, *p* = 0.007) and symbolic (2LV%, *p* = 0.006) analysis were significantly reduced in PD patients with GBA mutation compared to controls, while no significant differences emerged between controls and iPD. In supine position, GBA patients had also higher LF/HF values than the CTR group (*p* = 0.014). All the results are shown in Figure 2.

### Cardiovascular Autonomic Response to Orthostatic Challenge

**FIGURE 3:**
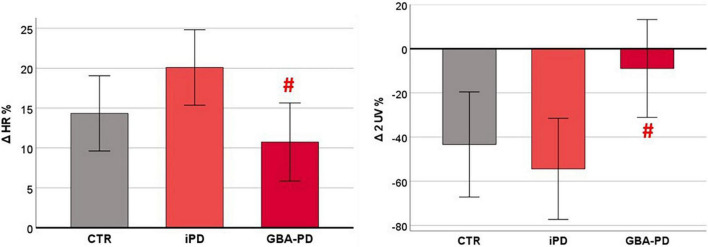
Differences between groups in cardiovascular autonomic response to orthostatic challenge. HR and HRV parameters are presented as estimated marginal means adjusted by Age. CTR, control group; iPD, idiopathic Parkinson’s Disease patients; GBA-PD, Parkinson’s Disease patients with GBA mutation; HR, heart rate; 2UV%, patterns with 2 unlike variations. # Significant *p*-values < 0.05, comparison versus iPD.

The statistical analysis on the orthostatic challenge response, evaluated through the Δ% values, revealed a significant difference between iPD and GBA-PD groups, as shown in Figure 3. In particular, patients carrying GBA mutation showed a lower increase of HR (*p* = 0.012) and a lower reduction of parasympathetic modulation (assessed by the 2UV% symbolic analysis index, *p* = 0.023) in response to the active standing test.

### Correlation Between Heart Rate Variability Indices at Rest and Clinical Features in Parkinson’s Disease Patients

**FIGURE 4:**
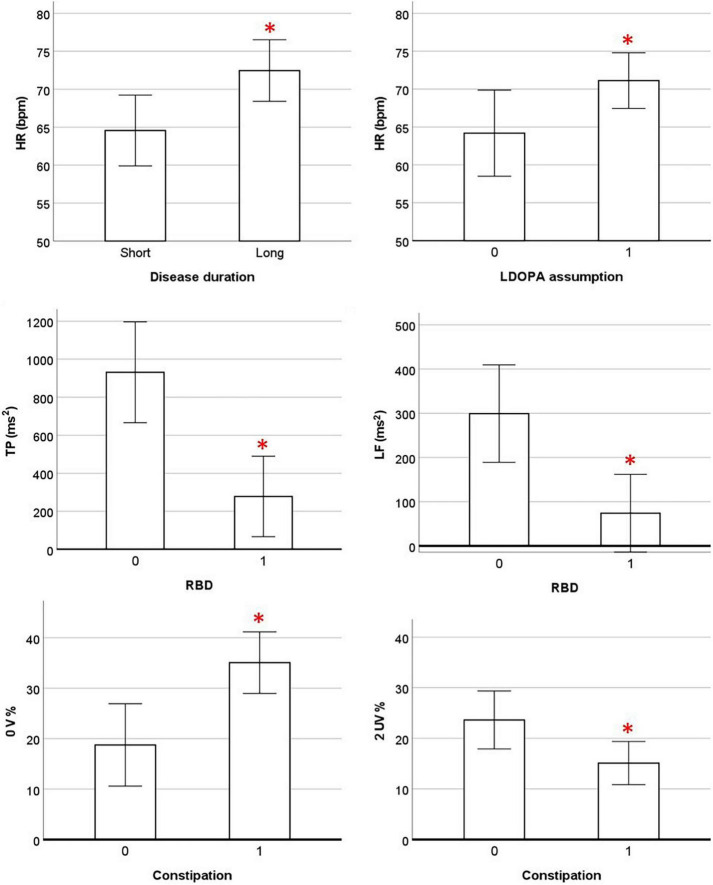
Association between cardiovascular parameters at rest and clinical data. HR and HRV parameters are presented as estimated marginal means adjusted by Age and presence of GBA mutation. 0, absence of symptom; 1, presence of symptom; HR, heart rate; LDOPA, levodopa; TP, total power; RBD, REM sleep behavior disorder; 0 V%, patterns with no variations; 2UV%, patterns with 2 unlike variations; OH, orthostatic hypotension. * Significant *p*-values < 0.05 in partial correlation analysis.

The partial correlation analysis between HRV indices at rest and clinical data was performed considering all PD patients (*N* = 30). The analysis was corrected for age and presence of GBA gene mutations. All the results are presented in Supplementary Material. A significant positive association was found between HR and disease duration (*r* = 0.388; *p* = 0.041). The TP and LF power were negatively associated with the presence of RBD (*r* = −0.582, *p* = 0.001 and *r* = −0.521, *p* = 0.004, respectively) and the occurrence of constipation symptoms was positively associated with 0V% (index of cardiovascular sympathetic modulation, *r* = 0.519; *p* = 0.005) and negatively associated with 2UV% (index of cardiovascular parasympathetic, *r* = −0.413; *p* = 0.029). No association was found between the severity of motor symptoms, phenotype and HRV indices. Finally, a positive trend close to significance (*r* = 0.368; *p* = 0.054) emerged between the assumption of levodopa therapy and HR. The significant associations between the cardiovascular autonomic control at rest and clinical data are shown in Figure 4.

## Discussion

The aim of our study was to objectively evaluate the differences in terms of cardiovascular autonomic control between a population of patients with iPD and a population of PD patients carrying mutations of the GBA gene. The observations from our study are of clinical significance with particular regard to the identification of a specific cardiovascular autonomic pattern: (i) both iPD and GBA-PD group were characterized by a lower spectral HRV; (ii) at rest, the GBA-PD group was characterized by higher HR values compared to the CTR group, resulting from a lower parasympathetic modulation and a shift of the sympathovagal balance toward a sympathetic predominance, as shown by both spectral and symbolic analysis; whereas the iPD group presented intermediate values between CTR and GBA-PD groups; (iii) the dynamic response of the CAC to the orthostatic challenge was significantly different in the two PD groups, as GBA-PD patients presented a lower HR increment and a lower or absent reduction of the vagal modulation; (iv) lastly, higher HR values were associated with longer disease duration and cardiovascular autonomic dysfunction was associated with the occurrence of RBD and constipation.

Previous reports suggested that the presence of a GBA mutations can have an impact on motor manifestations and progression, as well as on the development of cognitive impairment (Brockmann et al., 2011; Zhang et al., 2018; Maple-Grødem et al., 2021). Consistently with data shown in other cohorts (Goker-Alpan et al., 2008; Cilia et al., 2016; Petrucci et al., 2020), GBA-PD patients enrolled in this study presented more frequently an akinetic-rigid phenotype and cognitive disfunction, compared to iPD patients.

Dysautonomia is a well-known feature of PD that particularly contributes to impoverishing the patient quality of life and is often poorly controlled by current therapies. Neural apoptosis, α-synuclein accumulation and synaptic alterations were observed in central autonomic network areas, such as frontal cortex, hypothalamus and dorsal vagal nucleus (Braak et al., 2004; Muntané et al., 2008; Pablo-Fernandez et al., 2017), as well as in peripheral structures, such as vagus nerve, sympathetic nerve fibers, and enteric neural plexus (Braak et al., 2007; Probst et al., 2008; Gold et al., 2013; Wakabayashi, 2020). However, a centripetal progression of the disease has been suggested, with the autonomic nuclei of the spinal cord and the peripheral ANS representing the most constantly and earliest affected regions (Probst et al., 2008; Wakabayashi, 2020). Additionally, the analysis of HRV applied to autonomic cardiovascular tests has allowed over time to investigate in a non-invasive way the state and functional adaptability of the ANS to physiological stimuli in PD patients. To the best of our knowledge, this is the first study investigating HRV in GBA-PD patients at rest and in response to an orthostatic challenge.

A lower spectral HRV in iPD patients compared to healthy age-matched subjects was observed in several studies (Kallio et al., 2002; Soares et al., 2013; Strano et al., 2016) and confirmed by our data in PD patients, both with and without GBA mutations. As to the state of the sympathovagal balance at rest, we could observe for the first time a significantly reduced vagal modulation at rest in GBA-PD patients compared to healthy subjects, both through spectral and symbolic analysis. Moreover, as already reported by other authors [with the only exception of patients with longer disease duration (Mihci et al., 2006; Baumert et al., 2012; Strano et al., 2016; Vianna et al., 2016; Heimrich et al., 2021)], iPD patients did not show statistically significant differences compared to the CTR group, and were characterized by a phenotypically intermediate CAC, with values between CTR and the GBA-PD groups for all HRV indices.

In addition to the evaluation of the autonomic profile at rest, we also analysed the dynamic response of CAC to standing position, highlighting for the first time a significant difference between the two groups of PD patients. As a matter of fact, conversely to what was expected in the normal autonomic response to active standing, we observed a lower HR increase and blunted or absent vagal withdrawal in GBA-PD patients. Considering the response to orthostatic challenge, no great evidence of difference between iPD and related controls has been referred (Mihci et al., 2006; Strano et al., 2016; Vianna et al., 2016), except for patients with symptomatic OH and in advanced stages of PD (Haensch et al., 2009; Vianna et al., 2016).

As demonstrated by cellular models, the loss of GCase function directly interferes with α-synuclein degradation in lysosomes, while toxic aggregated α-synuclein inhibits normal lysosomal function of GCase (Murphy et al., 2014; Wildburger et al., 2020). This pathogenic loop may facilitate neurodegeneration in peripheral and central nervous system, resulting in earlier and more severe development of cardiovascular dysautonomia and cognitive disfunction, as shown in our study.

Even though different reports in literature suggest that cardiovascular dysautonomia correlates more with PD severity than with disease duration (van Dijk et al., 1993; Haapaniemi, 2001; Devos et al., 2003), we found higher HR values at rest in patients with longer disease duration. Higher resting HR values also characterized patients treated with levodopa, although this result did not reach the cut-off of significance. Acute levodopa administration has been shown to have negative inotropic but not chronotropic effects (Sénard et al., 1995; Noack et al., 2014). However, to the best of our knowledge, the effects of chronic levodopa therapy on CAC have not yet been investigated in detail.

It is known that sleep disturbances and sleep deprivation affect the capacity of the CAC to respond adaptively to internal and external stimuli (Takase et al., 2004; Tobaldini et al., 2013a,^2017^; Grimaldi et al., 2016), while ANS alterations can disturb the sleep-wake rhythm and the control of transitions between sleep stages (Tobaldini et al., 2013b; Miglis, 2016). Given these premises and since RBD is one of major non-motor predictors of α-synucleinopathies, with up to 65% risk of developing a neurodegenerative disease at 10 years from the onset of the sleep disorder (Postuma et al., 2012; Postuma and Berg, 2019), the relation between HRV and occurrence of RBD has been investigated. Consistently with other studies (Valappil et al., 2010; Postuma et al., 2011; Bugalho et al., 2018), our results showed that spectral HRV is negatively related to the occurrence of RBD in PD patients.

Among other potential prodromal symptoms of PD, constipation has been found to occur prior to motor manifestations in about 20 to 40% of cases and it has been estimated that an individual with chronic constipation has a 2.27-fold increased risk of developing PD than someone without (Adams-Carr et al., 2016; Knudsen et al., 2017; Picillo et al., 2021). From our analysis, patients who complained of constipation had higher sympathetic modulation and reduced vagal modulation, result that is part of a suggestive framework of enhanced overall autonomic deterioration in this specific subgroup of PD patients (Natale et al., 2013).

Our findings from partial correlation analysis may suggest underlying pathophysiological mechanisms common to different NMS that need to be investigated in larger cohorts of patients.

One of the main limitations of this study is the small sample size. Studies including age-stratified patients (from the onset to longer disease durations), as well as longitudinal studies are needed to assess over time the CAC of PD patients with GBA mutations, in order to clarify the dynamics of disease progression in this subgroup. The second limit is the lack of a complete evaluation of the dynamic autonomic response performed by other autonomic maneuvers (e.g., Valsalva maneuver, deep breathing, tilt test). A complete assessment could add information not only on the reaction to sympathetic stimulation but also on the reaction to vagal stimulation. Finally, in future studies, it will be necessary to evaluate and quantify NMS through specific questionnaires or objective measures in order to allow a more detailed description of symptom severity.

Despite some limitations, this is the first study that compares the cardiovascular autonomic control of PD patients with GBA mutations with that of iPD patients. In addition, the presence of the control group allows a direct comparison with reference values relative to healthy subjects.

## Conclusion

In conclusion, the present study suggests that GBA-PD patients present a severe cardiovascular autonomic dysfunction when compared to the control group, with a significant greater impairment of the parasympathetic component, and an altered dynamic response to the orthostatic challenge. These results, combined with a more careful profiling of non-motor symptoms in PD patients, could add new insights into the etiopathogenesis of autonomic impairment in α-synucleinopathies and lead to more effective therapeutic approaches. The present results suggest a different autonomic dysfunction profile within PD, based on the presence of GBA mutations. If further studies confirm the present findings, more attention will have to be paid from the clinical point of view on patients with PD carrying GBA mutations, outlining specific prevention programs for cardiovascular risk and planning more frequent monitoring with autonomic tests.

## Data Availability Statement

The raw data supporting the conclusions of this article will be made available by the authors, without undue reservation.

## Ethics Statement

The studies involving human participants were reviewed and approved by Comitato Etico Milano Area 2. The patients/participants provided their written informed consent to participate in this study.

## Author Contributions

AC, GL, AD, and ET: conceptualization and methodology. AC, GL, GR, GF, EM, FA, EF, IT, and PS: data curation. AC and GL: writing—original draft preparation. GR, GF, EM, FA, EF, IT, CB, LF, NM, AD, and ET: writing—review and editing. NM, AD, and ET: supervision. NM: funding acquisition. All authors have read and agreed to the published version of the manuscript.

## Conflict of Interest

The authors declare that the research was conducted in the absence of any commercial or financial relationships that could be construed as a potential conflict of interest.

## Publisher’s Note

All claims expressed in this article are solely those of the authors and do not necessarily represent those of their affiliated organizations, or those of the publisher, the editors and the reviewers. Any product that may be evaluated in this article, or claim that may be made by its manufacturer, is not guaranteed or endorsed by the publisher.
