# Supplementary material for: Dysautonomia in Parkinson’s Disease: Impact of Glucocerebrosidase Gene Mutations on Cardiovascular Autonomic Control
Source: Front Neurosci. 2022 Mar 15;16:842498. doi: 10.3389/fnins.2022.842498 (PMC8964968; doi:10.3389/fnins.2022.842498)
Supplement: Supplementary file 1 [file Table_1.DOCX]

Supplementary Material

**Supplementary Table 1.** Partial correlation analysis between HRV indices at rest and clinical features on PD patients (N=30).

| Control variables: group & age | | **Motor symptoms** | | | | | **Non-motor symptoms** | | | | | **Therapy** | | | | |
| --- | --- | --- | --- | --- | --- | --- | --- | --- | --- | --- | --- | --- | --- | --- | --- | --- |
|  |  | **Disease duration** | **H&Y** | **UPDRS III** | **Phenotype** | **Postural instability** | **RBD** | **Symptomatic OH** | **SH** | **Constipation** | **Urinary disturbs** | **Total LEDD** | **Levodopa assumption** | **Dopamine agonist assumption** | **Dyskinesias** | **Fluctuations** |
| **HR (bpm)** | r | **0.388** | 0.155 | 0.179 | 0.065 | 0.337 | 0.255 | -0.360 | 0.145 | 0.033 | -0.320 | 0.029 | **0.368** | -0.265 | -0.129 | 0.079 |
|  | *p* | ***0.041**** | *0.432* | *0.371* | *0.744* | *0.079* | *0.190* | *0.060* | *0.471* | *0.866* | *0.097* | *0.883* | ***0.054*** | *0.173* | *0.512* | *0.688* |
| **TP (ms^2^)** | r | -0.109 | -0.023 | 0.229 | -0.188 | -0.230 | **-0.582** | 0.042 | -0.277 | -0.087 | 0.046 | -0.083 | -0.096 | -0.148 | -0.051 | -0.133 |
|  | *p* | *0.580* | *0.908* | *0.251* | *0.338* | *0.240* | ***0.001**** | *0.831* | *0.161* | *0.661* | *0.816* | *0.675* | *0.626* | *0.453* | *0.795* | *0.499* |
| **LF (ms^2^)** | r | -0,108 | -0,029 | 0,347 | -0,300 | -0,304 | **-0,521** | 0,090 | -0,079 | -0,067 | 0,062 | -0,140 | -0,019 | -0,119 | -0,123 | -0,266 |
|  | *p* | *0,584* | *0,885* | *0,076* | *0,121* | *0,116* | ***0,004**** | *0,648* | *0,696* | *0,736* | *0,754* | *0,479* | *0,922* | *0,547* | *0,533* | *0,171* |
| **HF (ms^2^)** | r | 0,005 | 0,007 | 0,030 | -0,325 | -0,121 | -0,280 | -0,003 | -0,138 | -0,080 | -0,056 | -0,107 | -0,220 | -0,154 | 0,088 | -0,044 |
|  | *p* | *0,980* | *0,974* | *0,882* | *0,092* | *0,539* | *0,150* | *0,990* | *0,493* | *0,685* | *0,777* | *0,588* | *0,261* | *0,434* | *0,656* | *0,822* |
| **LF nu** | r | -0,050 | -0,129 | 0,099 | -0,360 | -0,031 | -0,058 | 0,083 | 0,079 | 0,037 | 0,082 | 0,012 | 0,036 | 0,157 | 0,020 | -0,126 |
|  | *p* | *0,801* | *0,514* | *0,623* | *0,062* | *0,876* | *0,768* | *0,675* | *0,694* | *0,851* | *0,678* | *0,952* | *0,857* | *0,425* | *0,921* | *0,521* |
| **HF nu** | r | 0,042 | 0,204 | 0,007 | 0,234 | -0,195 | -0,033 | -0,026 | -0,057 | 0,137 | 0,088 | -0,006 | -0,123 | 0,017 | 0,073 | 0,198 |
|  | *p* | *0,830* | *0,298* | *0,974* | *0,231* | *0,320* | *0,867* | *0,895* | *0,778* | *0,486* | *0,655* | *0,974* | *0,533* | *0,931* | *0,711* | *0,313* |
| **LF/HF** | r | 0,150 | -0,021 | 0,108 | -0,181 | 0,042 | 0,010 | -0,098 | 0,323 | -0,103 | -0,128 | 0,104 | 0,111 | 0,036 | 0,024 | -0,023 |
|  | *p* | *0,445* | *0,917* | *0,591* | *0,355* | *0,832* | *0,960* | *0,620* | *0,101* | *0,601* | *0,515* | *0,598* | *0,575* | *0,856* | *0,904* | *0,907* |
| **0V%** | r | 0.053 | 0.096 | -0.034 | -0.056 | -0.021 | -0.179 | -0.121 | 0.017 | **0.519** | -0.081 | 0.001 | 0.345 | -0.253 | 0.213 | 0.122 |
|  | *p* | *0.790* | *0.628* | *0.866* | *0.776* | *0.914* | *0.361* | *0.541* | *0.935* | ***0.005**** | *0.684* | *0.995* | *0.072* | *0.194* | *0.277* | *0.536* |
| **2LV%** | r | 0.065 | -0.258 | 0.054 | -0.177 | -0.096 | 0.089 | -0.238 | -0.308 | -0.208 | -0.016 | -0.115 | -0.240 | 0.056 | -0.194 | -0.142 |
|  | *p* | *0.741* | *0.185* | *0.788* | *0.369* | *0.628* | *0.651* | *0.223* | *0.118* | *0.288* | *0.935* | *0.559* | *0.218* | *0.778* | *0.323* | *0.472* |
| **2UV%** | r | 0.007 | 0.000 | 0.021 | 0.221 | 0.080 | 0.073 | **0.399** | 0.107 | **-0.413** | 0.284 | 0.235 | -0.105 | 0.147 | -0.042 | -0.036 |
|  | *p* | *0.972* | *1.000* | *0.916* | *0.259* | *0.688* | *0.711* | ***0.036**** | *0.594* | ***0.029**** | *0.143* | *0.230* | *0.594* | *0.455* | *0.833* | *0.856* |

Abbreviations: H&Y, Hohen and Yahr scale; UPDRS III, Unified Parkinson's Disease Rating Scale part III; RBD, REM sleep behavior disorder; OH, orthostatic hypotension; SH, supine hypertension; total LEDD, total levodopa equivalent daily dose; HR, heart rate; TP, total power; LF, low frequency; HF, high frequency; 0 V%, patterns with no variations; 2LV%, patterns with 2 like variations; 2UV%, patterns with 2 unlike variations. *Significant p values < 0.05.
